# Music assisted nitrous oxide oxygen inhalation sedation in pediatric dentistry: a prospective three-arm randomized clinical trial

**DOI:** 10.3389/fdmed.2026.1864735

**Published:** 2026-06-26

**Authors:** Bharati Honnekeri, Sundeep Hegde K., Sharan Sargod, Shailesh Ramdas Shenoy, Akib Sheikh

**Affiliations:** Department of Pediatric and Preventive Dentistry, Yenepoya Dental College, Yenepoya Deemed to be University, Mangalore, Karnataka, India

**Keywords:** dental anxiety, inhalation sedation, music therapy, nitrous oxide, paediatric dentistry

## Abstract

Nitrous oxide–oxygen inhalation sedation (NOIS) is the recommended first line pharmacological approach for managing dental anxiety in children. Music, owing to its psychoacoustic qualities and autonomic nervous system-influencing potential, emerges as a potential non-pharmacological agent in the management of dental anxiety. The aim of this study was to assess the efficacy of Indian instrumental music as an adjunct to nitrous oxide-oxygen Inhalation sedation. This prospective, three arm parallel- group randomized clinical trial was conducted at the Department of Pediatric and Preventive Dentistry, Yenepoya Dental College, Mangalore (CTRI/2024/04/065857). A total of 39 anxious child participants between the ages of 9 and 12 years (Mean age = 10.56 ± 1.14 years) who required restorative procedures under NOIS, as assessed by MCDASf were included in the study. They were equally distributed among the groups, i.e., Group A (Control Group), Group B (NOIS music through ambient speakers), and Group C (NOIS with music through earphones), each consisting of 13 participants. The intervention used was ‘Minuet in D major' from the album ‘Classicool' performed by sitar maestro Purbayan Chatterjee. Physiological responses such as systolic BP, diastolic BP, and pulse rate PR) were recorded. The earphone group (Group C) showed statistically significant change over time for all physiological parameters. *p* = 0.003, *p* = 0.011, *p* = 0.030, respectively, for systolic bp, diastolic bp, and PR. Intergroup comparisons revealed no statistically significant differences at baseline or post-intervention. A statistically significant between-group difference was noted during the intervention phase exclusively for PR (*p* = 0.014). The music intervention demonstrated high overall acceptability; 88.5% of participants reported liking the music and 84.6% indicated a desire for its continuation in future appointments (*p* = 1.000). The use of Indian instrumental music as an adjunct to NOIS in Earphone-delivered music demonstrated statistically significant within-group reductions in all physiological parameters, with a significant intergroup difference in pulse rate during intervention (*p* = 0.014). Most intergroup differences were non-significant, and these findings should be interpreted as clinically promising trends warranting larger randomized trials rather than definitive evidence of efficacy.

**Clinical Trial Registration:**

CTRI/2024/04/065857, https://ctri.nic.in/Clinicaltrials/searchbyctri.php

## 1 Introduction

Oral health in childhood has lasting consequences for systemic health, quality of life, and long-term patterns of dental attendance. A fundamental prerequisite for effective dental care in the paediatric population is the ability to manage the child's behaviour and anxiety sufficiently to allow treatment to be delivered safely and cooperatively. Dental anxiety is a complex state involving cognitive, emotional, and physiological components, ranging from mild apprehension to active resistance and complete treatment refusal (1, 2). Globally, between 5% and 24% of children and adolescents are estimated to experience clinically significant dental anxiety, with injection-related fear being the most consistently reported trigger (3, 4). If not managed, dental anxiety perpetuates a cycle of avoidance, deteriorating oral health, and reinforced negative dental experiences that frequently persists into adulthood (3).

Both pharmacological and non-pharmacological strategies are employed to manage dental anxiety in children. Behavioural methods such as the tell-show-do technique, positive reinforcement, and distraction are the mainstay of non-pharmacological interventions, while pharmacological interventions consist of conscious sedation, deep sedation, and general anaesthesia (5, 6). Amongst the pharmacological interventions, Nitrous Oxide-Oxygen Inhalation Sedation (NOIS) has been proven to be the gold standard for anxious child dental patients by the American Academy of Pediatric Dentistry (AAPD), which is considered valuable for its rapid onset of action, its ability to provide titratable anxiolysis with analgesic benefits, rapid post-procedural recovery, and a high safety profile (7, 8). Moreover, it has also been endorsed by other international organizations such as the European Academy of Paediatric Dentistry (EAPD) and the Italian Society of Paediatric Dentistry (SIOI) as the preferred option for a minimally invasive sedation technique for child dental patients (9, 10). However, it is also known that the effectiveness of NOIS is still limited in that it does not completely alleviate anxiety; rather, it depends on the severity of the anxiety, negative dental conditioning, temperament of the child, and a need for a safe and cost-effective adjunct that may help enhance its effectiveness (11).

One of the neurophysiological properties of nitrous oxide that has been relatively neglected in clinical literature is, the well-documented effect on auditory perception. As the effect of nitrous oxide on the other sensory modalities increases, the auditory system is the last to be impaired, and there is a corresponding increase in auditory acuity during the administration of the agent (12, 13). Background noise, including the vibratory noise of dental handpieces, suction devices, and air compressors, is perceived as amplified during the administration of NOIS, and this may have the effect of interfering with the relaxation that is the goal of the procedure (12, 14). This represents a particular window of clinical opportunity at which the modification of auditory perception, through the presentation of carefully selected music, may be seen to have the potential to be of direct benefit as a counterbalance to the environmental stimuli that may be contributing to the anxiety of the patient. Nitrous oxide has also been seen to have the effect of changing middle ear pressure and subjective sound intensity perception without affecting absolute auditory threshold levels, and this has direct implications for the method by which music is presented during the administration of the agent (14). It is particularly important to note that, although it might seem that earphones should be preferable over ambient speakers for the delivery of auditory stimuli for obvious reasons, no research had yet compared earphones and ambient speakers in the unique setting of NOIS, in which the physiological and psychological changes associated with nitrous oxide exposure would likely impact their effectiveness differently. The use of both forms of auditory stimuli delivery is thus an intentional design choice, based on a recognition of their unique qualities within NOIS.

Music has come to be increasingly recognized as a scientifically-supported non-pharmacological intervention with demonstrable effects on the autonomic, neurobiological, and psychological correlates of anxiety (15). By stimulating areas of the brain involved in emotion regulation, stress response, and autonomic nervous system functioning, such as the limbic system and hypothalamic-pituitary-adrenal axis, music has been shown to produce anxiolytic effects that are physiologically unique from pharmacological sedation but potentially complementary to it (16). Systematic reviews and meta-analyses have shown music interventions to be effective in decreasing anxiety-related physiological indicators such as heart rate and blood pressure in a variety of pediatric procedural and dental anxiety situations (17, 18). In addition, instrumental music, being free of lyrical distractions that could interfere with cognitive resources, has been shown to be particularly effective (15, 19).

The sitar was chosen as the instrument for use in this research based on a number of justifying reasons. To begin with, the psychoacoustic qualities of the sitar, namely the acoustic properties generated by the sitar, including resonance of the sympathetic strings due to their length, rich overtone content, and decay of tones characteristic of the instrument, are ideal for the induction of parasympathetic dominance associated with low levels of anxiety and autonomic downregulation (20). Furthermore, the sitar inherently possesses high cultural familiarity and positive valence for children in India, which would facilitate emotional and attentional responses to this type of stimulus. Finally, there is empirical evidence from Balan et al. (2009) showing that Indian classical instrumental music with a leading role played by the sitar significantly attenuates procedural pain during venepuncture procedures in paediatric patients (20). These properties, taken together, make the sitar a theoretically and empirically grounded choice as the instrumental vehicle for music-based anxiolysis in this study.

Despite this evidence, the adjunctive use of Indian instrumental music alongside NOIS in the paediatric dental setting has not been systematically investigated, and the influence of music delivery method on anxiolytic efficacy in this context remains entirely unexplored.

The present study was therefore designed to evaluate and compare the effect of Indian instrumental music, delivered via ambient speakers and via in-ear earphones, as an adjunct to NOIS on the physiological parameters of dental anxiety in children aged 9–12 years undergoing restorative dental treatment.

## 2 Methods

The study was conducted and reported in accordance with the CONSORT 2010 statement for randomised trials [Supplementary file 1].

### 2.1 Study design and setting

This prospective, three-arm, parallel-group clinical trial was conducted at the Department of Pediatric and Preventive Dentistry, Yenepoya Dental College, Mangalore, Karnataka, India. The study was prospectively registered with the Clinical Trials Registry – India (CTRI/2024/04/065857; registered 16 April 2024) prior to participant enrolment and was approved by the Yenepoya Ethics Committee-1, Yenepoya (Deemed to be University), Mangalore (Ref. No. IEC/YDC/2023/01/002). Written informed consent was obtained from the parent or legal guardian of each participant prior to enrolment, and oral assent was elicited from each child. The study was conducted in adherence with the Declaration of Helsinki.

### 2.2 Participants

Children aged 9–12 years attending the outpatient clinic requiring restorative dental treatment under NOIS were screened for eligibility against the following criteria.

Inclusion criteria: (1) age 9–12 years; (2) American Society of Anesthesiologists (ASA) physical status classification I or II; (3) confirmed dental anxiety on the Modified Child Dental Anxiety Scale Faces version (MCDASf); (4) requirement for restorative dental treatment not exceeding 30 min under NOIS; and (5) no prior exposure to NOIS.

Exclusion criteria: (1) inability to tolerate nasal mask placement; (2) history of epilepsy, seizure disorder, or current use of sedative medication; (3) sensory processing disorder, musculoskeletal disorder, or recent ear surgery; and (4) active upper respiratory tract infection or chronic obstructive pulmonary disease.

Participants were initiated to recruit for the study from 31-01-2025 and last recruitment was done on 06-01-2026.

Sample size was estimated using G*Power software (version 3.1), based on data from Marwah et al. (2005) (2), in which a clinically meaningful difference of 3 beats per min in pulse rate between music and control groups was reported, with a pooled standard deviation of 4. To detect this difference with 80% statistical power at a 10% level of significance, a minimum of 13 participants per group was required, yielding a total sample size of 39. It should be noted that this sample size was powered exclusively for the pulse rate; that study was therefore underpowered for SBP and DBP and for multiple interpreted with caution and regarded as hypothesis- generating rather than confirmatory.

### 2.3 Randomisation and allocation

Simple randomization was performed by participants drawing sequentially numbered chits (pre-labeled for each group, *n* = 13) from an opaque fish bowl, ensuring 1:1:1 allocation. The drawn chit was handed to the principal investigator, who recorded the allocation and proceeded accordingly. All clinical assessments and interventions were performed by the principal investigator to minimise inter-examiner variability. Blinding in allocation was ensured in the sense that the principal researcher did not know which chit would be chosen until the participant picked it. However, this simple process of chit picking poses the possibility of selection bias in small sample sizes because the labels in the other group will become more predictable as the recruitment process nears its end. The chances of selection bias were controlled by ensuring all the chits were labeled before the process of recruiting participants commenced and conducting the eligibility screening for all participants before allocation was made.

### 2.4 Interventions

All three groups received restorative dental treatment under NOIS as the standard of care. Participants in all groups were instructed to observe a *nil-per-os* (NPO) period of two hours prior to the procedure. Standard behavioural shaping using the tell–show–do technique was applied uniformly across all groups before sedation induction. NOIS was titrated to a sedation level of 4 on the Ramsay Sedation Scale, using a nitrous oxide concentration of 30%–50% administered via a nasal hood with scavenger system (Porter NOIS unit). Cavity preparation was performed using an air-turbine handpiece, and the prepared cavity was restored with glass ionomer cement (GlasIonomer FX Ultra Packable, A2 shade). One tooth was treated per participant.

Group A (control): Participants received NOIS alone, with no music intervention.

Group B (ambient speaker): Five minutes prior to sedation induction, Indian instrumental music was introduced via a Bluetooth ambient speaker (Sony SRS-XB13) positioned in the operatory at a volume of 75 dB (60% of maximum device output; Samsung Galaxy S22 Ultra).

Group C (earphone): Five minutes prior to sedation induction, Indian instrumental music was delivered via wireless in-ear earphones (OCCIAM T9) at the same volume setting (75 dB, 60% device output). Earphones were purchased by the principal investigator and were kept clean between participants. They were kept securely in place to remain so throughout the procedure.

This was an open label trial, as blinded participation was impossible due to the audible sound from the experimental condition, and blinded assessment could not occur since the interventionist performed the intervention and measured the variables as the main researcher. However, although the possibility of the performance bias and detection bias is obvious in this design, the major physiological variables used in this research (SBP, DBP, PR) are objective measures.

The music stimulus for Groups B and C was the same: a sitar performance of the “Minuet in D Major” from the album “Classicool” by Purbayan Chatterjee (Times Music, 2006). The music on this album falls into the category of fusion of Hindustani (North Indian) semi-classical music, which involves the reinterpretation of Western Baroque and classical music compositions through the Hindustani instrumental technique with the sitar being the main lead instrument. This is semi-classical music because of the relative ease of its melodies and relatively less complexity of its structure, thus making it suitable for a paediatric audience irrespective of their knowledge of Indian classical music. The music was kept on throughout the procedure and stopped after the last physiological recording. One standardized music track was specifically chosen for the study to control for between-subject differences in musical taste and provide equal stimulus exposure to all participants. This choice was guided by factors including the tempo of the music (between 60 and 80 beats per minute – a rhythm ideal for cardiovascular entrainment), cultural relevance for Indian children, and psychoacoustic qualities (resonance of the sitar that is favourable for parasympathetic activity). Music personalization was specifically avoided in this efficacy trial to assess the impact of the stimulus alone.

The outcome variables were systolic blood pressure (SBP), diastolic blood pressure (DBP), and pulse rate (PR), as objective measures of anxiety state. This was done by a calibrated digital arm sphygmomanometer (OMRON HEM-8712) and a pulse oximeter. The recordings were taken at three standardised time points: T1: pre-intervention before the induction of sedation; T2: during the procedure at the completion of the cavity preparation; and T3: post-intervention five minutes after the end of the sedation.

Secondary outcomes were the patient's acceptability of the music intervention, which was measured by two questions with a binary response format at the completion of the procedure. These questions were: ‘Did you like the music being played during the procedure?’ and ‘Would you like to listen to music again during your next appointment? Validation of the behaviourally-based or self-report anxiety scales (e.g., FLACC, Venham Scale) has not been done in this current study since the emphasis of the study was on the physiological measures of anxiety in the presence of NOIS based on the existing sedation literature where the physiological indicators are highly valued. The questions about acceptability are meant to gauge preferences rather than psychological tests; validated multi-modal self-report anxiety assessment will be explored further in the future.

### 2.5 Statistical analysis

Summary statistics were computed for the variables using mean and standard deviation for continuous variables and frequency and percentage for categorical variables. Normality of distribution was assessed using the Shapiro–Wilk test. As several variables demonstrated non-normal distribution (*p* < 0.05), non-parametric tests were employed throughout. Within-group changes in SBP, DBP, and PR across the three time points were assessed using the Friedman test. Between-group differences at each time point were evaluated using the Kruskal–Wallis test. Patient acceptability responses were compared between delivery groups using Fisher's exact test. A *p*-value of less than 0.05 was considered statistically significant. All analyses were performed using IBM SPSS Statistics version 22.0.

## 3 Results

### 3.1 Participant characteristics

The study enrolled 39 children aged 9–12 years (mean age 10.56 ± 1.14 years), of whom 22 were male (56.4%) and 17 were female (43.6%). Thirteen participants were allocated to each group. The distribution of age and sex across the three groups was comparable, with no statistically significant baseline differences observed. The Shapiro–Wilk test indicated non-normal distribution for several outcome variables (*p* < 0.05); accordingly, non-parametric tests were used for all subsequent analyses.

### 3.2 Within-group changes in physiological parameters

Friedman test analysis revealed that children listening to music through ambient speakers (Group B) showed no meaningful change in any physiological parameter across the three time points. In contrast, children in the earphone group (Group C) demonstrated statistically significant reductions across all three parameters — systolic BP, diastolic BP, and pulse rate, with a consistent progressive decline observed from the pre-intervention through the procedural and post-intervention phases. Group A (control), which received no music intervention, showed no consistent directional trend across time points for any parameter; descriptive values are presented in Table 1 for reference, and within-group Friedman analysis was not applicable to this group as no intervention was administered (Table 1).

**Table 1:** Within-group comparison of SBP, DBP, and PR across time points (friedman test).

Parameter	Group	Pre mean (SD)	During mean (SD)	Post mean (SD)	*χ* ^2^	*p*-value
Systolic BP (mmHg)	Group A (Control)^a^	98.54 (13.08)	98.69 (15.42)	95.38 (12.55)	—	N/A
	Group B (Speaker)	94.77 (6.22)	94.08 (5.88)	94.92 (5.66)	1.755	0.416
	Group C (Earphone)	97.15 (10.38)	94.69 (8.92)	94.15 (9.75)	11.792	0.003*
Diastolic BP (mmHg)	Group A (Control)^a^	72.77 (7.92)	72.69 (10.25)	70.69 (8.73)	—	N/A
	Group B (Speaker)	70.38 (4.79)	69.15 (6.72)	70.00 (5.89)	0.275	0.872
	Group C (Earphone)	68.00 (5.96)	66.46 (4.65)	66.15 (4.91)	8.974	0.011*
Pulse Rate (bpm)	Group A (Control)^a^	89.00 (13.31)	91.77 (13.45)	88.46 (12.46)	—	N/A
	Group B (Speaker)	82.92 (8.47)	82.08 (4.43)	82.31 (5.62)	0.480	0.787
	Group C (Earphone)	82.92 (8.60)	78.92 (7.61)	79.54 (7.95)	7.000	0.030*

Within-group changes in Systolic BP, Diastolic BP, and Pulse Rate across pre-, during-, and post-intervention time points for Group B (Speaker Music) and Group C (Earphone Music). Friedman test used. *p < 0.05 considered statistically significant. Green cells denote significant *p*-values.

a Friedman test was not applied to Group A (Control) as no music intervention was administered. Descriptive values are included for completeness and for reference in between-group comparisons (Table 2).

### 3.3 Between-group comparisons

The three groups were well-matched at baseline across all physiological parameters, confirming comparability prior to intervention. Table 2 presents group-wise mean ± SD values for all three groups (Group A: Control, Group B: Speaker, Group C: Earphone) at each time point alongside the Kruskal–Wallis test statistics, making the three-group nature of the comparison transparent. During the active treatment phase, systolic and diastolic BP did not differ meaningfully between groups; however, pulse rate showed a statistically significant divergence, suggesting that the mode of music delivery had a differential effect on heart rate during the procedure. By the post-intervention assessment, these differences had largely resolved, with no parameter reaching statistical significance, though the pulse rate comparison came close (Table 2).

**Table 2:** Between-group comparison of SBP, DBP, and PR at each time point (Kruskal–Wallis test, df = 2).

Time point	Parameter	Group A mean (SD)	Group B mean (SD)	Group C mean (SD)	Overall mean (SD)	H statistic (df = 2)	*p*-value
Pre-intervention	Systolic BP	98.54 (13.08)	94.77 (6.22)	97.15 (10.38)	96.82 (10.14)	1.072	0.585
	Diastolic BP	72.77 (7.92)	70.38 (4.79)	68.00 (5.96)	70.38 (6.49)	2.105	0.349
	Pulse Rate	89.00 (13.31)	82.92 (8.47)	82.92 (8.60)	84.95 (10.51)	1.256	0.534
During intervention	Systolic BP	98.69 (15.42)	94.08 (5.88)	94.69 (8.92)	95.82 (10.75)	0.594	0.743
	Diastolic BP	72.69 (10.25)	69.15 (6.72)	66.46 (4.65)	69.44 (7.81)	4.350	0.114
	Pulse Rate	91.77 (13.45)	82.08 (4.42)	78.92 (7.61)	84.26 (10.60)	8.505	0.014*
Post-intervention	Systolic BP	95.38 (12.55)	94.92 (5.66)	94.15 (9.75)	94.82 (9.50)	0.153	0.926
	Diastolic BP	70.69 (8.73)	70.00 (5.89)	66.15 (4.91)	68.95 (6.84)	2.321	0.313
	Pulse Rate	88.46 (12.46)	82.31 (5.62)	79.54 (7.95)	83.44 (9.66)	5.817	0.055

Between-group comparison of Systolic BP, Diastolic BP, and Pulse Rate among all three groups (Group A: Control, Group B: Speaker, Group C: Earphone) at pre-, during-, and post-intervention time points. Kruskal–Wallis test used (df = 2). *p < 0.05 statistically significant.

### 3.4 Music acceptability

Music was well-received regardless of delivery method, with the large majority of children across both music groups reporting that they enjoyed the music and would welcome it at a future appointment. No significant difference in acceptability was found between the speaker and earphone conditions, indicating that children found both modes equally agreeable. The results are similar to the acceptability rates reported by Marwah *et al*. and Aitken *et al*. (2, 21). It is important to note that the high acceptability of both methods (88.5% liking the music in general; 84.6% desiring the continuity of the intervention) provides a basis for the clinical implementation of musical interventions in pediatric dentistry irrespective of the mode of delivery. It can be said that the lack of differences in the objectively measured indices despite high subjective acceptability of the intervention is a significant observation in terms of clinical practice as it means that even without any physiological impact, children may enjoy music during procedures (Table 3).

**Table 3:** Music liking and preference for next appointment by delivery method (Fisher's exact test).

Outcome	Group B — Speaker (*n* = 13)	Group C — Earphone (*n* = 13)	*p*-value
Liked the music	11 (84.6%)	12 (92.3%)	1.000
Opted for music at next appointment	10 (76.9%)	12 (92.3%)	0.593
Overall (both groups combined): 88.5% liked music; 84.6% opted for music at next visit			

Association between mode of music delivery (speaker vs. earphone) and children's liking of music and preference for music at the next appointment. Fisher's exact test used. *p* > 0.05 = not significant (NS).

## 4 Discussion

The present study investigated, for the first time, the adjunctive effect of Indian instrumental music on physiological markers of dental anxiety in children receiving restorative treatment under NOIS, and specifically compared two modes of music delivery ambient speakers and earphones against a no-music control. The primary finding was that earphone-delivered Indian instrumental music produced statistically significant progressive reductions in all three physiological parameters of anxiety systolic BP, diastolic BP, and pulse rate across the procedural timeline, while ambient speaker delivery produced a consistent but non-significant downward trend in the same parameters. A statistically significant between-group difference in pulse rate during the intervention phase further confirmed that the three groups diverged meaningfully during active treatment. These findings are discussed below in the context of the existing literature.

### 4.1 Music as an anxiolytic adjunct to NOIS

The anxiolytic properties of music in pediatric dental settings are well-supported by the existing evidence base. Systematic reviews and meta-analyses by Gokhale et al. (2024) and Ting et al. (2022) have consistently documented that music interventions produce significant reductions in physiological stress markers, including heart rate and blood pressure, across a range of procedural and dental contexts in children (22, 23). Individual clinical trials have reported comparable findings: Jindal et al. demonstrated significant anxiety reduction with audio distraction during both restorative and invasive pediatric dental procedures, while Gupta et al. reported improved cooperation and reduced anxiety scores in children receiving music as a non-pharmacological adjunct (24, 25). The directional trend observed in the present study with both music groups showing lower physiological parameter values relative to the control group at all time points is consistent with this body of evidence and supports the biological plausibility of music-induced anxiolysis as an adjunct to NOIS.

This synergy has a strong mechanistic foundation. NOIS exerts its anxiolytic and analgesic effects through GABAergic central nervous system pathways, reducing affective and cognitive processing of fear stimuli (8, 26). In the induction phase of NOIS, nitrous oxide causes dose-dependent suppression of visual and somatosensory evoked potentials without any effect on brainstem auditory evoked potential latencies, thus suggesting a differential and hierarchical depression of sensory modalities in which the auditory pathway is preferentially functionally preserved (27). Music, by contrast, acts through complementary and anatomically distinct pathways, stimulating limbic and subcortical regions involved in emotional regulation, autonomic modulation, and stress response attenuation, while simultaneously engaging cortical attentional resources through the distraction mechanism (15, 16). The two interventions thus address anxiety at pharmacological, autonomic, and cognitive levels simultaneously, offering a plausibly synergistic rather than merely additive effect. The absence of statistical significance in the between-group comparison overall is most parsimoniously attributed to the limited sample size of 13 participants per group, a constraint determined by power calculations based on the primary physiological endpoint rather than to the absence of a true effect, particularly given the statistically robust within-group findings in the earphone group.

The most clinically novel finding of this study is the superior anxiolytic efficacy of earphone delivery over ambient speaker delivery, a comparison that has not previously been reported in the context of NOIS.

Three converging mechanisms offer a coherent explanation for this difference.

First, N₂O produces measurable changes in middle ear pressure and subjective sound intensity perception during sedation, as demonstrated by Fabijan et al. and Tekavec (12, 14). These physiological alterations may impair the fidelity of sound reaching the middle ear via ambient speakers, as the signal must traverse an altered middle ear environment. Earphones, which deliver sound directly to the external auditory meatus, bypass middle ear pressure changes entirely, ensuring consistent and undegraded auditory input throughout the duration of sedation. This distinction is particularly relevant in the context of the current study, as the NOIS-enhanced auditory acuity documented in the sedation literature means the quality of the auditory stimulus may have a disproportionate influence on the patient's experience during sedation compared with non-sedated conditions (12).

Second, the clinical dental operatory is characterised by a continuous background of anxiety-provoking auditory stimuli that are well-established triggers of anxiety in the paediatric dental patient (28, 29). Earphones attenuate these ambient stressors by passive acoustic occlusion, effectively replacing aversive environmental sound with the chosen musical stimulus. Ambient speakers deliver music into a sound field already occupied by operatory noise, reducing both the signal-to-noise ratio and the cognitive distraction efficacy.

Third, the acoustic characteristics of the sitar rich sympathetic string resonance, complex overtone profiles, and sustained tonal decay are subject to significant degradation in the presence of ambient noise and at moderate room speaker volumes. Direct earphone delivery preserves the full harmonic integrity of the instrument's output, potentially enhancing both aesthetic engagement and the physiological entrainment effect of the music.

The selection of *Minuet in D Major* from Purbayan Chatterjee's *Classicool* album as the musical stimulus warrants specific discussion. The cardiovascular entrainment hypothesis of music therapy proposes that the human autonomic nervous system tends to synchronise with a regular, consistent external auditory rhythm particularly stimuli in the 60–80 beats per minute range gradually shifting the balance toward parasympathetic dominance and producing measurable reductions in heart rate and blood pressure (26, 30). The Minuet's moderate triple-metre structure and regular phrase periodicity make it well-suited to this mechanism, and its tempo falls within the entrainment-optimal range.

Musical stimulus selection, specifically the selection of “Minuet in D Major” from Purbayan Chatterjee's “Classicool” music album, is worthy of particular discussion. The cardiovascular entrainment theory suggests that the human autonomic nervous system has a tendency to entrain, or become synchronized, with a consistent, predictable external auditory rhythm, particularly a rhythm within the range of 60–80 beats per minute, slowly shifting the balance towards parasympathetic dominance, thereby resulting in a measurable decrease in heart rate and blood pressure (26, 30). The musical stimulus selected, “Minuet in D Major,” is a moderate tempo, triple-meter musical piece that is consistent in its periodicity. The selected musical stimulus for the present study, ‘Classicool' by Purbayan Chatterjee, belongs to the category of North Indian semi-classical fusion that uses elements of Baroque and Classical Western music, but within the idiomatic context of North Indian instrumental tradition. This blend allows easy melodic access to Indian children without any previous experience with Indian classical music, yet maintaining enough cultural connection and psychoacoustics of the sitar's sympathetic string resonance that may be involved in anxiety reduction (31).

The overall music acceptability observed in this study was found to be remarkably high: 88.5% of the children reported a preference for the music, and 84.6% liked the music. This is closely consistent with the findings observed by Marwah et al. and Aitken et al. (2, 21). The acceptability was found to be non-significantly different between the speaker and the earphone groups (*p* = 1.000 and *p* = 0.593, respectively), indicating the dissociation between the subjective and the objective effects.

However, certain limitations need to be taken into consideration. Although the sample size for this study was 13 for each group, which is sufficient for the primary outcome measure for this study according to *a priori* power calculations, the sample size may have been inadequate for the evaluation of the other outcome measures. The design of the study is a limitation; the use of an open allocation randomisation method and the impossibility of blinding the participants to the intervention may cause an expectation bias. Although SBP, DBP, and PR are objective outcome measures, they are only indirect measures of the anxiety state and may be affected by other factors. Individual variability in musical preference, familiarity with Indian instrumental music, and cultural background could not be entirely standardised across participants. Some of the confounding factors that should be noted include: The differences between subjects' anxiety levels at the beginning of the study were controlled by using the MCDASf test as a selection criterion; nonetheless, some differences may have existed since there was variability within the acceptable levels of anxiety in the selected group of patients. Previous dental experience was kept to a minimum through the exclusion of those who had been subjected to NOIS before; nonetheless, previous dental experience as such remained uncontrolled. Noise in the operatory was not recorded, although standardized conditions of the same room were kept for all patients.

## 5 Conclusions

This randomized clinical trial of Indian instrumental music as a supplement to NOIS in anxious children aged 9–12 years old, which measured dental anxiety by comparing earphone and ambient music presentation and a no-music control. However, the findings indicated that intergroup comparisons were not significant for any of the measurements made, apart from the intervention period pulse rate, which proved to be significant (*p* = 0.014). The findings may thus be considered as showing promising trends clinically rather than being conclusive proof of superiority. The use of earphones with Indian instrumental music showed significant intra-group improvements in all anxiety biomarkers studied, suggesting further studies involving multicentre randomized trials measuring behaviourally and subjectively measured anxiety.

## Data Availability

The raw data supporting the conclusions of this article will be made available by the authors, without undue reservation.
