# Supplementary material for: Music assisted nitrous oxide oxygen inhalation sedation in pediatric dentistry: a prospective three-arm randomized clinical trial
Source: Front Dent Med. 2026 Jun 26;7:1864735. doi: 10.3389/fdmed.2026.1864735 (PMC13350019; doi:10.3389/fdmed.2026.1864735)
Supplement: Supplementary file 1 [file Datasheet1.pdf]

Figure 1: CONSORT 2025 Flow Diagram

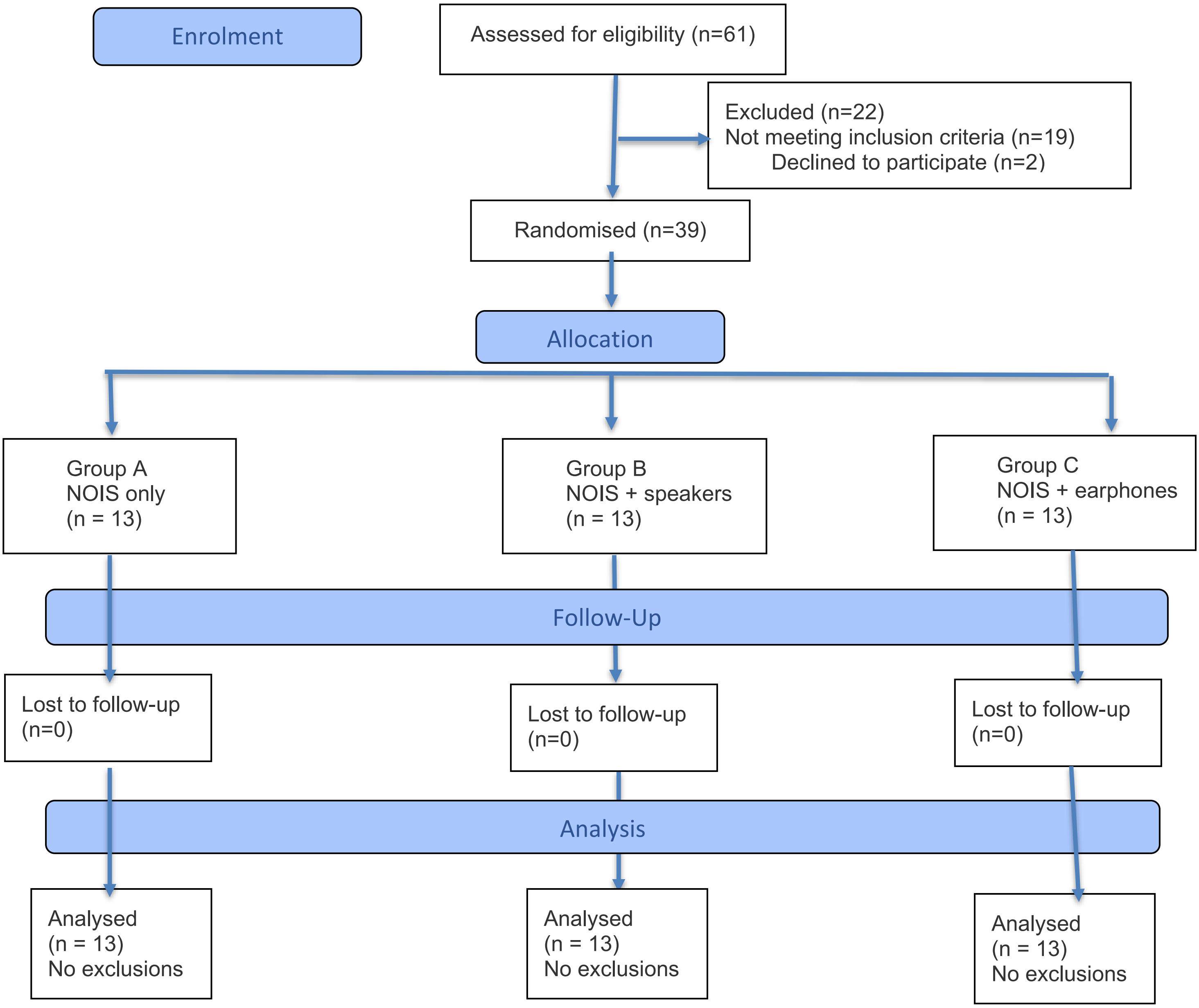

Citation: Hopewell S, Chan AW, Collins GS, Hróbjartsson A, Moher D, Schulz KF, et al. CONSORT 2025 Statement: updated guideline for reporting randomised trials. BMJ. 2025; 388:e081123. https://dx.doi.org/10.1136/bmj-2024-081123
© 2025 Hopewell et al. This is an Open Access article distributed under the terms of the Creative Commons Attribution License (https://creativecommons.org/licenses/by/4.0/), which permits unrestricted use, distribution, and reproduction in any medium, provided the original work is properly cited.
